# Evaluation of nanoencapsulated bevacizumab combined with paclitaxel in a colorectal cancer xenograft model

**DOI:** 10.1007/s13346-025-01941-6

**Published:** 2025-08-14

**Authors:** Cristina Pangua, Socorro Espuelas, Jon Ander Simón, María Collantes, Iván Peñuelas, Alfonso Calvo, Juan M. Irache

**Affiliations:** 1 Department of Pharmaceutical Sciences, School of Pharmacy and Nutrition, NANO-VAC Research Group, University of Navarra, Pamplona, 31008 Spain; 2 Program in Solid Tumors, CIMA of the University of Navarra, Pamplona, 31008 Spain; 3 Department of Chemistry, University of Navarra, Pamplona, 31008 Spain; 4 Radiopharmacy Unit, Clinica Universidad de Navarra, Pamplona, 31008 Spain; 5 Translational Molecular Imaging Unit (UNIMTRA), Department of Nuclear Medicine, Clinica Universidad de Navarra, Pamplona, 31008 Spain; 6 Institute for Health Research (IdiSNA), Pamplona, 31008 Spain

**Keywords:** Bevacizumab, Paclitaxel, Albumin nanoparticles, Dextran, Colorectal cancer

## Abstract

The combination of bevacizumab and paclitaxel (PTX) has exhibited synergistic antitumor effects in different types of solid cancer tumors probably due to a potential increase of intratumoral PTX facilitated by the action of the antiangiogenic agent bevacizumab. The aim of this study was to evaluate the benefits of using nanoencapsulated bevacizumab (B-NP-DEX) in combination with paclitaxel as part of a systemic therapeutic regimen for colorectal cancer (CRC). For this purpose, bevacizumab (either as aqueous solution or nanoencapsulated in albumin based-nanoparticles coated with dextran) in combination with paclitaxel were intravenously administered and evaluated in a xenograft mouse model of CRC. Both treatments (free and nanoencapsulated bevacizumab and PTX) significantly reduced tumor growth, metabolic activity, and weight, extending tumor-doubling time by approximately 5 days when compared with control animals (receiving saline). However, animals treated with the combination between PTX and B-NP-DEX showed enhanced antitumor effects than the conventional treatment with free bevacizumab, including a greater reduction in tumor growth rate, VEGF levels, and proliferation markers. PET imaging and biodistribution analysis revealed an 8-fold higher tumor-to-plasma ratio for the nanoencapsulated bevacizumab, suggesting improved tumor targeting via the enhanced permeability and retention effect. These findings support the potential of nanoparticle-based delivery systems to increase therapeutic efficacy while potentially reducing systemic toxicity, warranting further investigation into their role in optimizing antiangiogenic chemotherapy.

**Graphical abstract:**

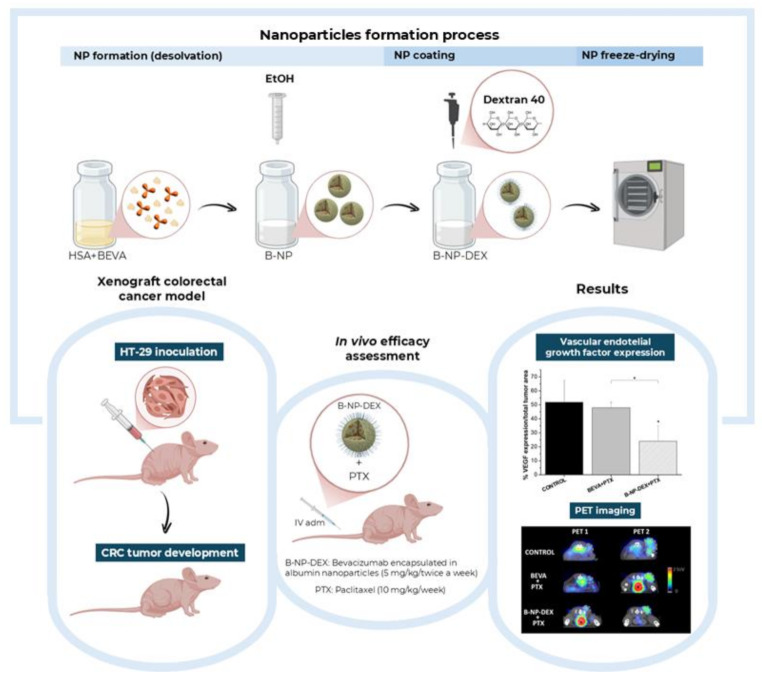

**Supplementary Information:**

The online version contains supplementary material available at 10.1007/s13346-025-01941-6.

## Introduction

Bevacizumab belongs to a relatively newer class of cancer therapeutics known as angiogenesis inhibitors. As a monoclonal antibody (mAb), bevacizumab selectively targets and neutralizes vascular endothelial growth factor (VEGF), a crucial protein involved in promoting new blood vessels formation that supports tumor growth and metastasis [1]. By inhibiting angiogenesis, bevacizumab effectively deprives tumors of their blood supply, hindering their expansion and ultimately leading to regression [2, 3].

The clinical history of bevacizumab in the past 20 years has raised concerns about its safety. Several adverse effects have been reported, including hypertension, gastrointestinal perforations, and even therapy resistance, all of which limit the effectiveness of bevacizumab [4–6]. Despite these facts, bevacizumab is still a leading treatment option. Its antiangiogenic effect has been reported to contribute to the normalization of tumor vascularization, enhancing the efficacy of the chemotherapeutic agents administered concurrently [7]. In this context, the addition of bevacizumab to oxaliplatin-based chemotherapy significantly improved progression free survival (PFS) as first-line trial in patients with metastatic colorectal cancer [8]. In a metanalysis of randomized controlled trial involving patients with metastatic breast cancer, bevacizumab significantly increased PFS with different chemotherapy regimens [9]. Moreover, the combination of bevacizumab and paclitaxel was evaluated in a MX-1 human breast cancer xenograft model where bevacizumab treatment significantly increased the concentration of paclitaxel in the tumor [10].

Paclitaxel (PTX), a well-established chemotherapeutic agent derived from taxanes, plays a pivotal role in the treatment of various cancer types, including breast, ovarian, lung, and pancreatic cancers [11, 12]. Its unique chemical structure consists of a C-13 side chain attached to the taxane ring, which imparts potent antitumor properties compared to other taxanes [13]. PTX exerts its therapeutic effects by disrupting the process of cell division, leading to apoptosis through its ability to bind and stabilize microtubules [14].

Furthermore, the clinical efficacy of PTX is often limited by adverse effects such as hypersensitivity reactions, myelosuppression, and peripheral neuropathy, which are frequently attributed to the excipients used in its formulation [15, 16]. For this reason, an alternative could be the combination of PTX with other chemotherapeutic or adjuvants to enhance its therapeutic efficacy [17, 18].

The combination of PTX and bevacizumab has provided higher efficacy and survival benefits in at least breast [19, 20], ovarian [21, 22], cervical [23], and peritoneal cancer [24]. The synergistic effect achieved by PTX and bevacizumab is due to different complementary mechanisms and leads to improved treatment efficacy. In a multicentre observational study, patients with HER2-negative metastatic breast cancer who received PTX plus bevacizumab as first-line chemotherapy, has a significantly better overall survival (OS) and PFS than those receiving PTX alone [25]. Tao and co-workers also reported that the addition of bevacizumab to PTX plus carboplatin in advanced cervical cancer, increased the treatment response and OS; although the treatment was not well tolerated [26]. Moreover, a significant increase in PFS and OS by the addition of bevacizumab to weekly PTX was observed in epithelial ovarian cancer [27].

In this context, the aim of this study was to evaluate the benefits of using nanoencapsulated bevacizumab (previously evaluated as a single treatment in a published study [28]) in a in combination with paclitaxel as part of a systemic therapeutic regimen for colorectal cancer (CRC). For this purpose, bevacizumab (either as aqueous solution or nanoencapsulated in albumin based-nanoparticles coated with dextran) in combination with paclitaxel were intravenously administered and evaluated in a xenograft mouse model of CRC.

## Materials and methods

### Materials

Reagents including human serum albumin (HSA), dextran (35,000–45,000) (DEX), sodium chloride, DMEM, sucrose, phenol, TRIS base, TBS, Tween^®^ 20, EDTA, sodium deoxycholate, and SDS were obtained from Sigma-Aldrich (Steinheim, Germany). Bevacizumab (Avastin^®^) was purchased from Roche (Madrid, Spain) and paclitaxel (Paclitaxel Hospira 6 mg/mL) from Hospira (Lake Forest, IL, USA). Ethanol was supplied by Scharlab (Sentmenat, Spain), and the Shikari^®^ Q-Beva ELISA kit from Matriks Biotek (Gölbaşı, Turkey). PBS tablets came from Medicago (Uppsala, Sweden), and sodium hydroxide from Honeywell (Charlotte, NC, USA). Isoflurane (IsoVet^®^) was purchased from B. Braun Vetcare (Rubí, Spain). Panreac AppliChem (Castellar del Vallès, Spain) provided HCl, formaldehyde, sulfuric acid, hematoxylin, and xylene. 2-deoxy-2-[^18^F]fluoro-D-glucose (^18^F-FDG) was produced by the Department of Nuclear Medicine and PET, University of Navarra. FBS, L-glutamine, and antibiotics were from Lonza (Basel, Switzerland). Antibodies were sourced from NeoMarkers (VEGF), Cell Signaling Technology^®^ (Caspase-3), and Thermo Fisher Scientific (Ki67). Secondary antibodies and DAB + system were from DAKO (Glostrup, Denmark), and DPX mounting medium from Merck (Darmstadt, Germany). All other materials were analytical grade.

### Preparation of human serum albumin nanoparticles coated with dextran

Human serum albumin (HSA) nanoparticles, coated with dextran 40,000 (DEX), were prepared as described previously [28], with minor modifications. Briefly, nanoparticles were obtained from an aqueous solution of albumin and bevacizumab at pH 6.4 after addition of ethanol. The resulting nanoparticles were incubated with 500 µL of a dextran solution (100 mg/mL) before purification by centrifugation and freeze-drying using sucrose as crioprotectant. This formulation was identified as B-NP-DEX.

### Physico-chemical characterization of nanoparticles

B-NP-DEX formulation was resuspended in water for injection at a concentration of 2 mg/mL to determine the size and zeta potential of the nanoparticles in a ZetaPlus analyser system (Brookhaven Instruments Corporation, Holtsville, USA).

The amount of HSA in the resulting nanoparticles (yield) was quantified using high-performance liquid chromatography (HPLC) as described before [28] and the payload of bevacizumab in the nanoparticles was quantified by ELISA following the manufacturer’s instructions. In both cases, previous to the analysis, 10 mg of the formulation were dispersed in 0.025 N NaOH and agitated for 3 min at room temperature before dilution in water for injection for analysis.

Dextran conjugation to nanoparticles was quantified via the phenol-sulphuric acid assay, as described by Zeng et al. [29]. Nanoparticles were purified by centrifugation at 41,000 *× g* for 20 min at 4 °C. Aliquots of 180 µL from both the resuspended pellet and corresponding supernatant were each mixed with 900 µL of 96% sulfuric acid, followed by the addition of 180 µL of 5% (w/v) phenol solution. Samples were incubated at 90 °C for 15 min and subsequently cooled in an ice bath for 5 min. Absorbance was measured at 490 nm using a PowerWave XS UV–VIS microplate reader (BioTek Instruments, USA) to determine dextran content.

### Animals

Athymic nude female mice (4–6 weeks old) were purchased from Envigo (Indianapolis, USA). All the animal experiments were under strict guidelines evaluated and approved by Ethical and Biosafety Committee for Research on Animals (University of Navarra), following the European legislation on animal experimentation (protocol 090 − 22). Xenograft colorectal tumor model was stablished by injecting subcutaneously in the right lateral flank 2.5 × 10^6^ HT-29 tumor cells (bevacizumab-sensitive human colon cancer cell line).

### In vivo antitumor efficacy

**Fig. 1:**
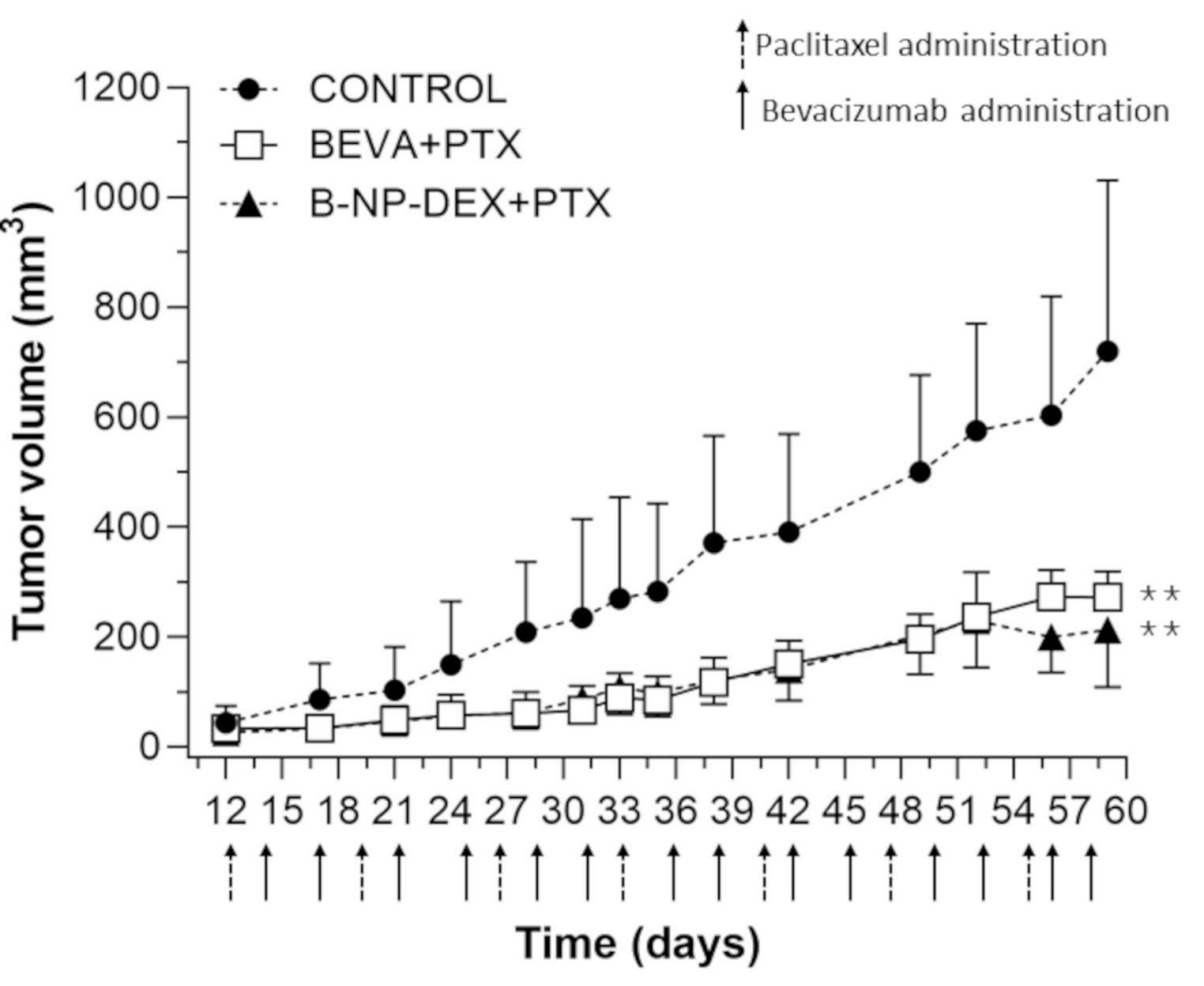
Tumor volume evolution in a mouse model of CRC for the different treatments tested. (i) BEVA + PTX: intravenous combination of a solution of bevacizumab with paclitaxel; (ii) B-NP-DEX + PTX: intravenous combination of nanoencapsulated bevacizumab in DEX-coated nanoparticles (B-NP-DEX) and paclitaxel. Bevacizumab (either free or nanoencapsulated) was administered intravenously at a dose of 5 mg/kg/ twice a week. Intravenous paclitaxel was administered at a dose of 10 mg/kg once a week. Data expressed as mean ± SD, (*n* ≥ 6). **p < 0.01 compared to control

Mice were divided randomly into three groups when the tumor volume reached approximately 50 mm^3^. The first group served as the control and received an intravenous injection of saline. The second group was treated with a combination of bevacizumab (diluted in water for injection) and PTX (prepared by dilution in saline solution). The third group received dextran-coated albumin nanoparticles loaded with bevacizumab (B-NP-DEX) and PTX saline solution. Bevacizumab groups (free or encapsulated in nanoparticles) received an iv dose of 5 mg/kg of body weight twice per week. Intravenous PTX was administered at a dose of 10 mg/kg of body weight once a week separately from bevacizumab administration. The control group received iv injections of saline at the same time points than the treated groups. A diagram summarizing the efficacy study is shown in the Supplementary material section (Fig. 1S). 

Tumor dimensions were measured throughout the study using a digital caliper in two orthogonal directions: width (W) and length (L). The tumor volume (V, in mm³) was estimated as the product of the squared width and the length, divided by 2

1 $$Tumor{\text{ }}volume\, = \,\left( {widt{h^2}\, \times \,length} \right)\,/\,2$$

To assess tumor progression, the tumor doubling time (DT), defined as the number of days required for the tumor volume to double, was calculated using Eq. 2 [30]:

2 $$DT\, = \,\left( {tf\,--\,{t_o}} \right)\, \times \,ln\left( 2 \right)\,/\,ln\left( {Vf\,/\,{V_o}} \right)$$

where Vf is the final tumor volume at time tf, and V_0_ is the initial volume at time t_0_.

The relative tumor volume (RTV), defined as the ratio between the tumor volume at a given time point and the volume at the beginning of treatment, was used to normalize tumor growth over time. Finally, the tumor growth inhibition (TGI) was calculated as the different in percentage between the RTV of the treated and control groups.

### Tumor activity by PET imaging

Tumor metabolism was assessed in vivo on days 15 and 42 from the beginning of the administration using [^18^F]-FDG PET imaging. Mice were fasted overnight with free access to water, anesthetized with 2% isoflurane in 100% O_2_, and injected intravenously with 11.2 ± 1.4 MBq of [^18^F]-FDG (80–100 µL). After 1 h of tracer uptake, 15-minute scans were acquired using a MicroPET tomograph (Mosaic, Philips, USA) in the prone position. Images were reconstructed via 3D RAMLA (two iterations, relaxation parameter 0.024) into a 128 × 128 matrix (1 mm voxel), with corrections for dead time, decay, scatter, and randoms. CT scans (U-SPECT6/E-class, MILabs) were also acquired for anatomical reference. PET data were analyzed using PMOD software, with [^18^F]-FDG uptake expressed as Standardized Uptake Value (SUV). SUV was calculated as the quotient of tissue radiotracer uptake and the injected dose, multiplied by body weight [31].

Tumor regions were manually defined using CT guidance, followed by semi-automatic segmentation of voxels exceeding 50% of SUV_max_. Three metabolic parameters were calculated to evaluate the tumor metabolic activity [31]: (i) the highest voxel activity- SUV_max_, (ii) the metabolic tumor volume (MTV, expressed in cm^3^), and (iii) the total lesion glycolysis (TLG), calculated as the product of MTV and SUV_mean_.

### Quantification of bevacizumab in plasma and tumor tissues

Blood samples were obtained weekly from the submandibular vein of mice. After collection, the samples were centrifuged at 5,000 rpm for 10 min, and the resulting plasma was stored at -80 °C until further analysis. Bevacizumab levels in plasma were determined using the Shikari^®^ Q-BEVA ELISA kit, following the manufacturer’s protocol. Absorbance was measured at 450/650 nm using a PowerWave HT Microplate Reader (BioTek Instruments, Inc., Winooski, USA).

To quantify bevacizumab within tumor tissues, samples were mechanically homogenized using a chilled tissue grinder (Biospec, Bartlesville, USA). The ground tissue was suspended in 300 µL of RIPA buffer to facilitate protein extraction. The homogenates were kept on ice for 30 min and centrifuged at 16,100 × g for 30 min at 4 °C. The supernatants were analyzed by ELISA to measure bevacizumab content. In parallel, total protein concentration was determined using the microBCA protein assay kit.

### Tumor analysis

Formalin-fixed paraffin-embedded tumor blocks were fixed in paraffin. Sections were then cut and dyed with haematoxylin-eosin staining. After that, tumor sections were visualized at 20x magnification acquiring the images on a Histological Slide Scanner Leica Aperio CS2 (Leica Biosystems, Wetzlar, Germany). Tumor tissue and necrotic areas of the tumor were differentiated via the image analysis software QuPath.

For immunohistochemical quantification of cleaved caspase-3, vascular endothelial growth factor (VEGF), and Ki67, paraffin-embedded tumor sections were deparaffinized and rehydrated and treated with 10% H_2_O_2_ for 10 min to block endogenous peroxidase. Antigen retrieval was performed at 95 °C for 20 min in Tris-EDTA buffer (pH 8) for VEGF and cleaved caspase-3, or in citrate buffer using a PT Link system (DAKO) for Ki67. Slides were washed with TBS-T and incubated overnight at 4 °C with primary antibodies: cleaved caspase-3 (1:200), VEGF (1:50), and Ki67 (1:200). After washing, sections were incubated for 30 min at RT with species-specific HRP-conjugated secondary antibodies. Detection was done using DAB+, followed by counterstaining with Harris hematoxylin. Slides were dehydrated, cleared in xylene, and mounted in DPX. Images were captured at 20x using a Leica Aperio CS2 scanner and analyzed with QuPath software.

### Statistical analysis

The means and standard errors were calculated for every data set. All the group comparisons and statistical analyses were performed using a one-way ANOVA test followed by a Tukey-Kramer multicomparison. Dunnett’s multiple comparison test was performed for ^18^F-FDG tumor uptake and immunohistochemistry analysis. In all cases, *p* < 0.05 was considered as a statistically significant difference. Significant differences are marked as follows: *p < 0.05, **p < 0.01 or ***p < 0.001. All calculations were performed using GraphPad Prism v6 (GraphPad Software, San Diego, CA, USA) and the curves were plotted with the Origin 8 software from Origin Lab (Origin Lab Corp, Northampton, MA, USA).

## Results

### Characterization of bevacizumab-loaded nanoparticles

Bevacizumab loaded nanoparticles exhibited a mean particle size of approximately 255 nm and a negative zeta potential of − 36 ± 5 mV. The amount of dextran was estimated in about 40 µg/mg nanoparticles. The encapsulation efficiency of bevacizumab was around 88%, with a drug loading capacity of approximately 110 µg of bevacizumab per mg nanoparticles.

### Tumor size progression

The effect of combining bevacizumab with PTX, either in its free form or encapsulated in nanoparticles, was evaluated using a colorectal cancer xenograft model. The results are shown in Fig. 1, which represents the changes in tumor volume (measured in mm³) over the course of the treatment period.

**Fig. 2:**
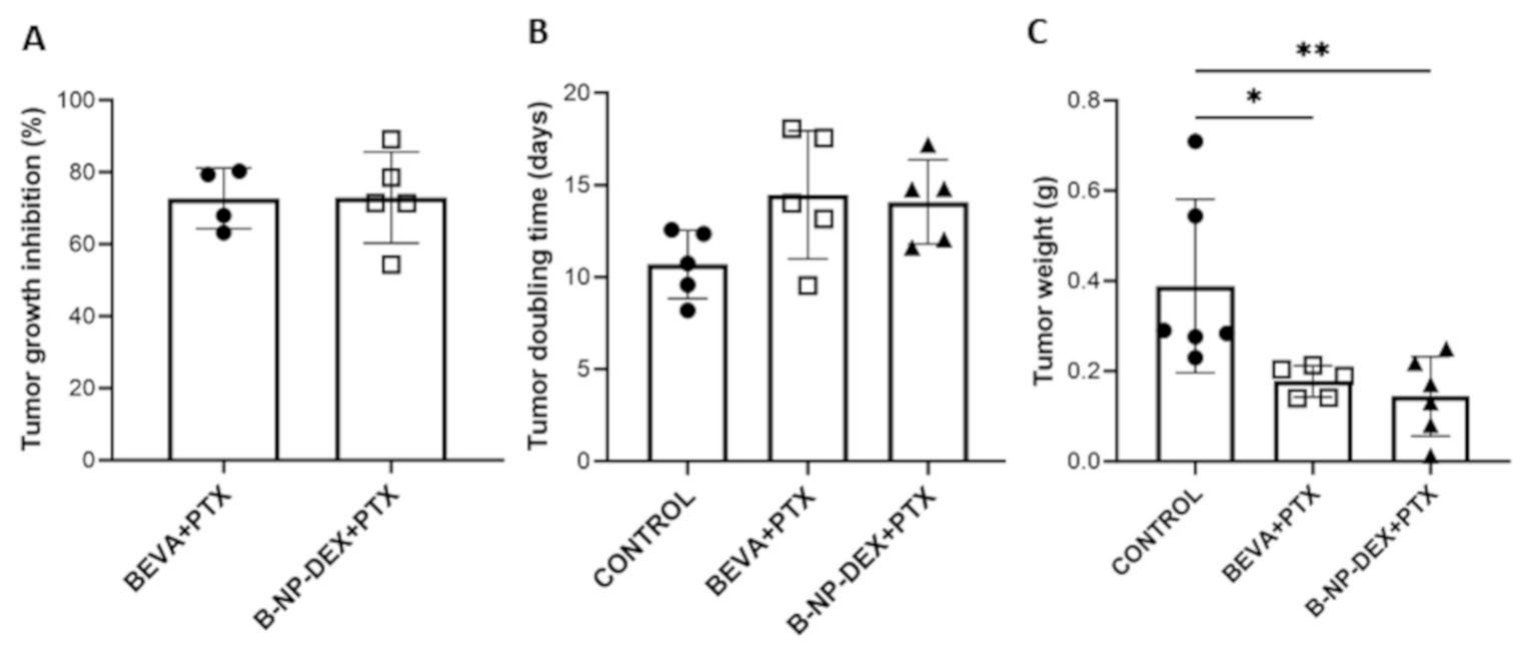
**(A)** Representation of the tumor growth inhibition rate (TGI) at the end of the study. **(B)** Tumor volume doubling time of the different treatments evaluated. **(C)** Tumor weight of the different treatments evaluated. Data expressed as mean ± SD, (*n* ≥ 4). *p < 0.05, **p < 0.01 compared to control

By the end of the study, both treatment groups showed a significant reduction in tumor volume compared to the control group. However, the group treated with bevacizumab-loaded nanoparticles (B-NP-DEX), in combination with intravenous PTX, exhibited a notably higher tumor growth inhibition; approximately a 70% reduction compared to the control group. The tumor growth inhibition rate, normalized to the control group, was about 60% for all the treatment groups (Fig. 2A). No statistically significant differences between the therapeutic strategies (BEVA + PTX vs. B-NP-DEX + PTX) were found. Regarding the tumor doubling time, this parameter was about 30% higher (approx. 5 days) in tumors of animals treated with any type of combination between bevacizumab and PTX than for those of control animals (Fig. 2B). At the end of the study, the tumors were extracted from the animals and weighed (Fig. 2C). The tumors of animals treated with any of the combinations between bevacizumab and PTX displayed a significant lower weight than those of control animals. However, B-NP-DEX + PTX showed a higher reduction compared to control group (*p* < 0.01).

**Fig. 3:**
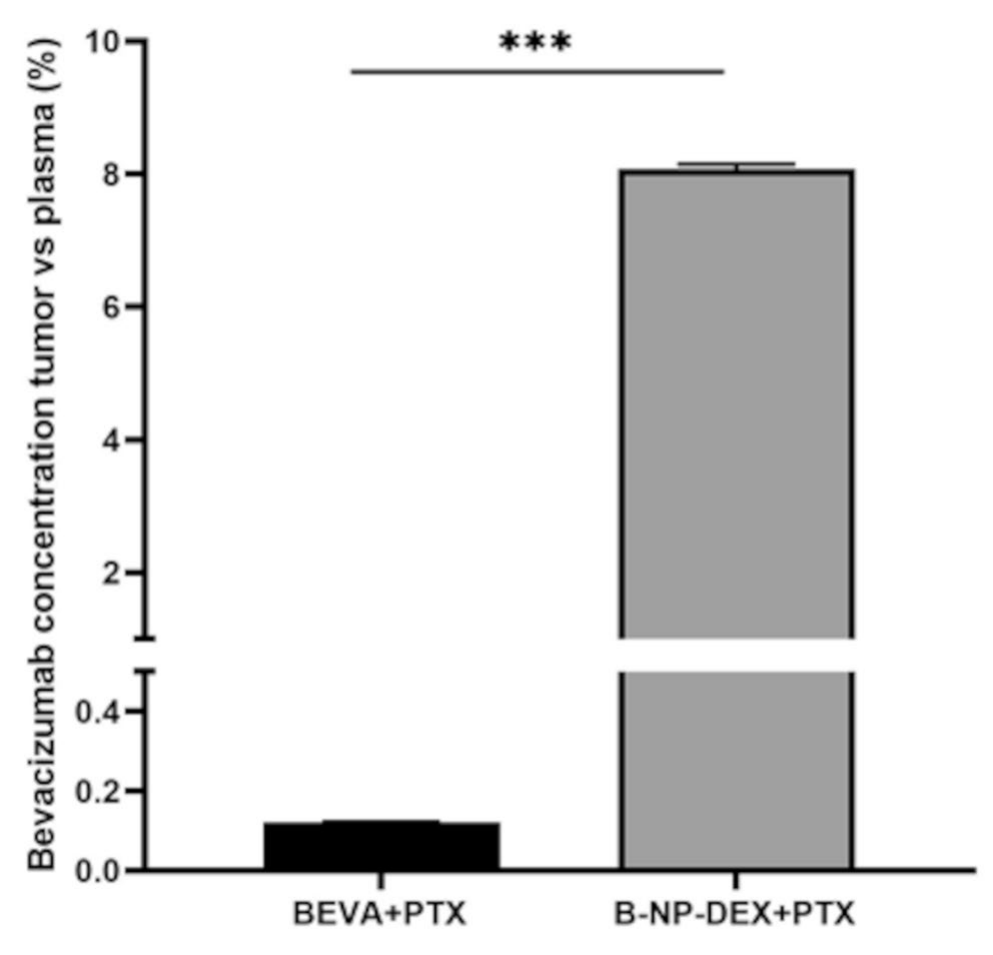
Bevacizumab concentration in tumor versus plasma in animals treated with either free bevacizumab (BEVA) or nanoencapsulated bevacizumab (B-NP-DEX) in combination with free paclitaxel (PTX). Data expressed mean ± SD, (*n* ≥ 4). ***: *p* < 0.001

### Bevacizumab quantification

Bevacizumab concentrations in plasma and tumor tissues were quantified using ELISA. Tumor-to-plasma ratios revealed a marked increase in intratumoral bevacizumab accumulation, up to 8-fold higher, in the B-NP-DEX + PTX group compared to the free BEVA + PTX treatment, indicating significantly enhanced tumor targeting and retention (Fig. 3).

### PET analysis

**Fig. 4:**
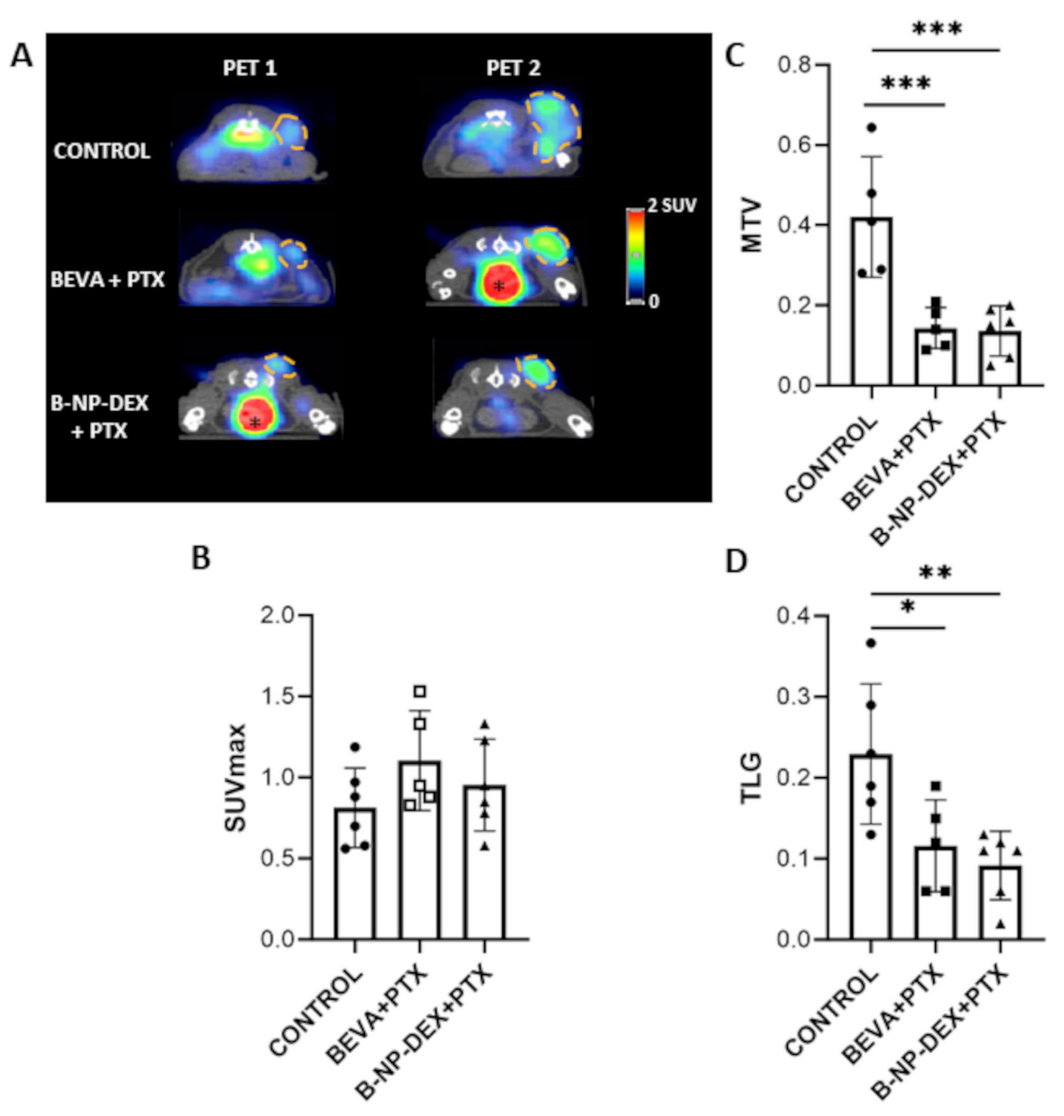
**(A)** PET images showing tumor uptake (dashed circles) for the different treatment groups: control, intravenous bevacizumab and paclitaxel (BEVA + PTX), and intravenous bevacizumab encapsulated into DEX-coated nanoparticles and paclitaxel (B-NP-DEX + PTX). **(B)** SUV_max_: maximum standardized uptake at day 42; **(C)** MTV: metabolic tumor volume at day 42; **(D)** TLG: total lesion glycolysis day 42. Data are expressed as mean ± SD, (*n* ≥ 6). *p < 0.05; **p < 0.01; ***p < 0.001 vs. control

The uptake of ^18^F-FDG in tumors was evaluated by PET imaging following a single dose administration. Scans were performed at two time points: day 15 (PET-1; data not shown) and day 42 (PET-2) from the beginning of treatment. As shown in Fig. 4A, PET images revealed uptake of ^18^F-FDG by tumors, particularly in control animals and with animals treated with B-NP-DEX + PTX showing the lowest radioactivity intensity.

Quantitative analysis of the microPET images focused on three key parameters: maximum standardized uptake value (SUV_max_, Fig. 4B), metabolic tumor volume (MTV, Fig. 4C), and total lesion glycolysis (TLG, Fig. 4D). No significant differences in SUV_max_ were observed across the treatment groups. However, considering the volume of interest (VOI), the analyses revealed significant differences in MTV and TLG. Specifically, MTV-2 values were significantly lower in both combination groups (BEVA + PTX and B-NP-DEX + PTX) compared to the control group (*p* < 0.01). In a similar way, the TLG values were also significantly lowers in animals treated with BEVA + PTX (*p* < 0.05) and B-NP-DEX + PTX (*p* < 0.01) than in controls.

### Tumor analysis

**Fig. 5:**
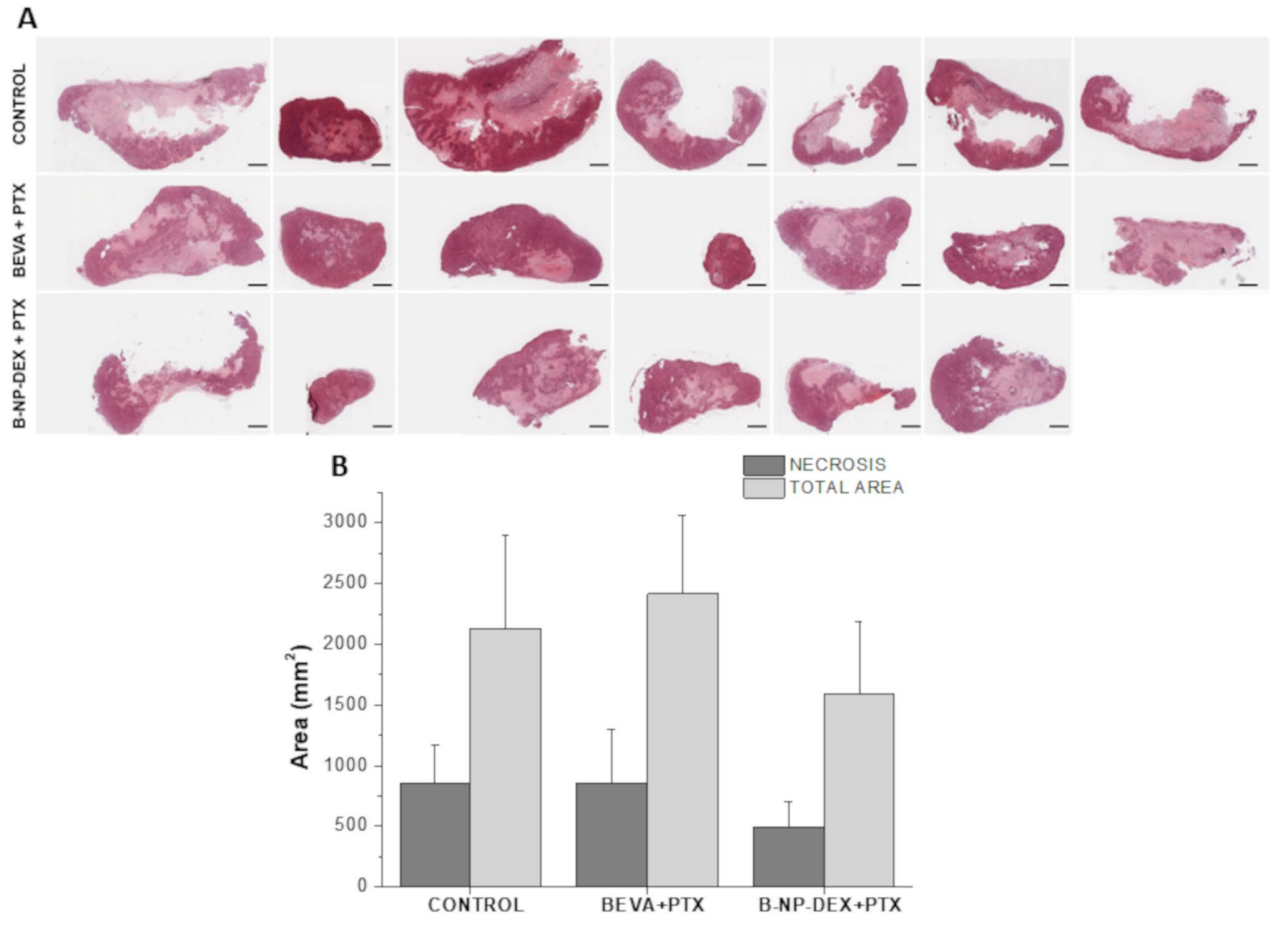
**(A)** Haematoxylin eosin-stained tumor sections of the different groups of animals: control, intravenous bevacizumab and paclitaxel (BEVA + PTX), and intravenous bevacizumab encapsulated into DEX-coated nanoparticles and paclitaxel (B-NP-DEX + PTX). **(B)** Necrotic and total tumor areas of the different treatments in mm^3^. Data are expressed as mean ± SD, (*n* ≥ 6)

Tumor necrotic areas were assessed via haematoxylin-eosin (H-E) staining. H-E-stained tumor sections revealed distinct necrotic regions, mainly located in the tumor core, characterized by pale-staining zones indicative of loss of cellular integrity and presence of cellular debris (Fig. 5A). Quantitative analysis of the stained sections by image analysis showed varying degrees of necrotic tissue among treatment groups. Although no statistically significant differences were observed compared to the control group (Fig. 5B), total tumor areas appeared slightly to significantly reduced in animals treated with the combination of PTX and nanoencapsulated bevacizumab, compared to control or free BEVA + PTX groups.

**Fig. 6:**
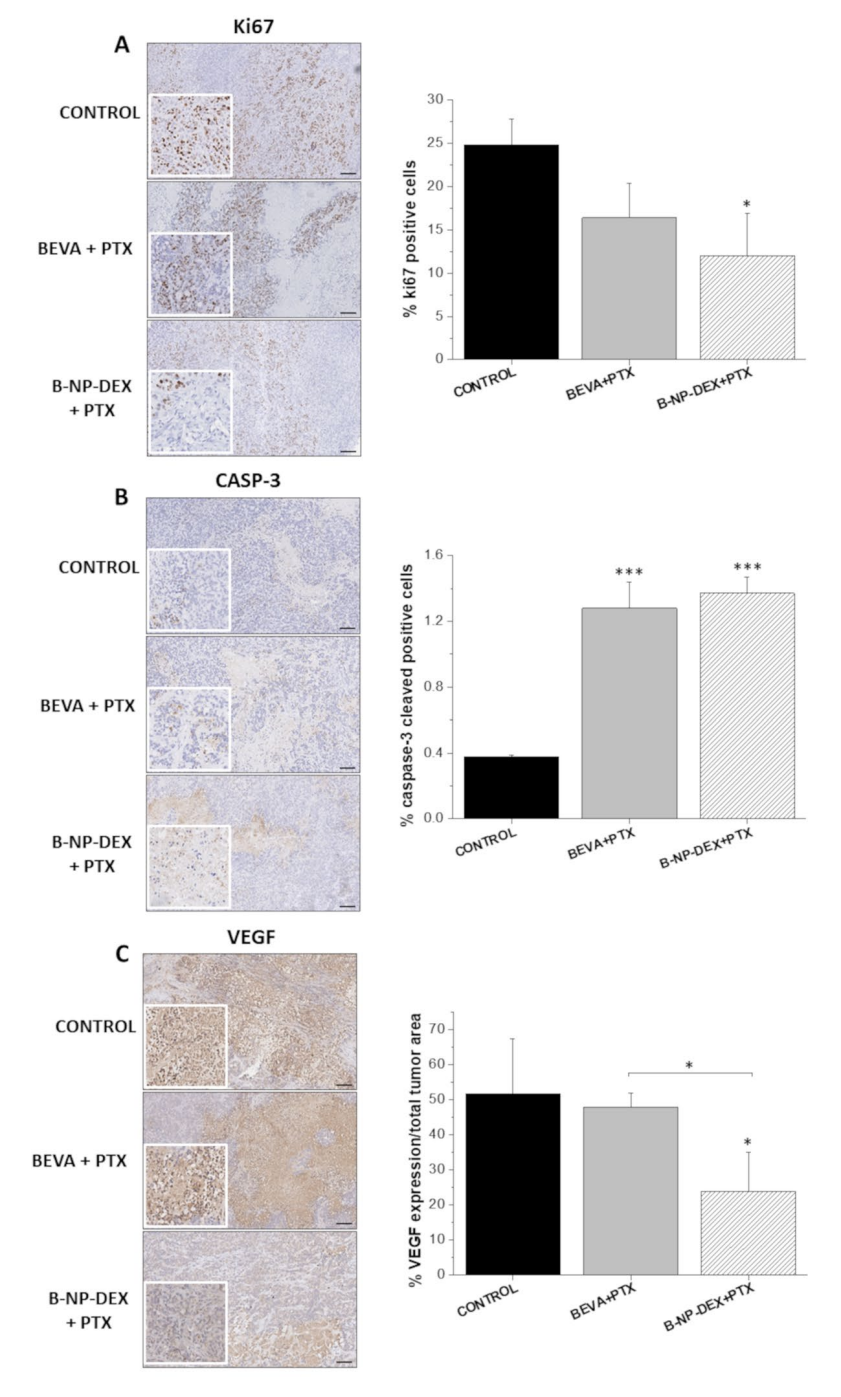
IHC staining of tumor sections for Ki67, cleaved caspase-3, and VEGF in the different groups of animals: control, intravenous bevacizumab and paclitaxel (BEVA + PTX), and intravenous bevacizumab encapsulated into DEX-coated nanoparticles and paclitaxel (B-NP-DEX + PTX). Scale bar represents 100 μm. **(A)** Percentage of Ki67 positive cells. **(B)** Percentage of caspase-3 cleaved positive cells. **(C)** Vascular endothelial growth factor area. Data are expressed as mean ± SD (*n* ≥ 3). *p < 0.05; ***p < 0.001 compared to control. T-student was used to compare BEVA + PTX vs. B-NP-DEX + PTX in VEGF expression

Immunohistochemistry (IHC) was performed to evaluate the expression and localization of cleaved caspase-3, Ki67, and VEGF in tumor tissues. IHC images revealed distinct staining patterns corresponding to the respective proteins of interest (Fig. 6). Quantitative analysis of the IHC staining was performed to quantify the expression levels of the biomarkers. Ki67 expression was evaluated as a marker of tumor proliferation [32]. Figure 6A shows a significant reduction in proliferation activity (Ki67; *p* < 0.01) was observed in tumors from mice treated with B-NP-DEX + PTX compared to controls.

Moreover, the expression of cleaved caspase-3 (an active form of capase-3) was evaluated as a key enzyme involved in the execution phase of apoptosis, or programmed cell death. In all groups, this enzyme was significantly increased in animals treated with the combination between bevacizumab (either free or nanoencapsulated) and paclitaxel than in animals receiving saline (Fig. 6B). Finally, VEGF expression, a marker of angiogenesis and neovascularization, was significantly reduced in the B-NP-DEX + PTX group compared to both the BEVA + PTX group and the control (Fig. 6C).

## Discussion

Inside the different types of solid tumors, colorectal cancer is situated in the top 3 most diagnosed and deadliest cancer [33]. The appearance of systemic toxicity from current treatments regimens, coupled with unsatisfactory response rates and the emergence of drug resistance, significantly hampers the prospects of a positive prognosis for CRC patients [34, 35]. Hence, considerable efforts have been made for the development of innovative approached that can either refine or entirely replace the existing CRC chemotherapy methods [36, 37]. The pursuit of novel therapeutics avenues promises to not only enhance treatment efficacy but also improve the overall patient’s quality of life.

One such strategy involves the combination of paclitaxel and bevacizumab. Paclitaxel has demonstrated efficacy in the treatment of both recurrent and metastatic colorectal cancer [38] and is commonly administered alongside other agents such as fluorouracil, oxaliplatin, leucovorin, and irinotecan. Bevacizumab, an anti-VEGF monoclonal antibody, inhibits angiogenesis by targeting vascular endothelial growth factor, thereby inducing regression of abnormal tumor vasculature, modulating vascular function, and promoting normalization of tumor blood flow [39, 40]. This vascular remodeling can lead to a reduction in the elevated interstitial fluid pressure (IFP) typically observed in tumors, which otherwise hinders the effective delivery and distribution of low molecular weight chemotherapeutic agents [41, 42]. Anti-VEGF therapies like bevacizumab have been shown to induce vascular normalization, facilitating the formation of a hydrostatic pressure gradient across the tumor vasculature. This, in turn, enhances drug penetration and improves therapeutic efficacy [43–46]. Yanagisawa et al. reported that the combination of paclitaxel (PTX) and bevacizumab exhibited synergistic antitumor effects in a xenograft breast cancer model, potentially due to increased intratumoral PTX concentrations associated with reduced vascular permeability [10]. In line with this, Cesca and collaborators demonstrated that the treatment with bevacizumab ameliorated the tumor distribution of paclitaxel, particularly in proliferating areas, improving its therapeutic activity. This effect was found independently of the tumor model or their site of implantation, and the different pattern of vascular response after antiangiogenic treatment [47].

Taking all these factors into account, the aim of this study was to evaluate the synergistic effect of PTX and bevacizumab in a subcutaneous colorectal cancer tumor model. In this approach, bevacizumab was encapsulated in albumin-based nanoparticles and administered in combination with PTX. This combinatorial strategy aimed to assess whether nanoparticle-mediated delivery could enhance the intratumoral accumulation of bevacizumab, thereby improving the overall therapeutic response to paclitaxel. Polymeric nanoparticles, such as those based on PLGA, have been widely used for the encapsulation of mAbs, including bevacizumab, leading to increased retention time and improved stability of the mAb in various pathological contexts [48–50]. However, there is growing interest in exploring alternative materials, such as proteins, for the encapsulation of mAbs in nanoparticle systems Notably, the incorporation of albumin into PLGA nanoparticle formulations has been shown to protect bevacizumab, primarily through protein interfacial adsorption [51]. As the body’s primary transport protein, albumin plays a critical role in drug delivery (i.e. high drug-loading capacity, controlled drug release) [52]. While albumin-based nanoparticles have been employed for the encapsulation of various anticancer agents (most prominently paclitaxel, marketed as Abraxane^®^), their use for mAb encapsulation remains relatively underexplored. Nevertheless, albumin offers unique properties that make it a promising carrier for such therapeutic agents, particularly in the context of anti-tumor therapies.

Although nanoparticle formulations can boost the enhanced permeability and retention (EPR) effect, albumin itself has been shown to improve this effect independently [53]. However, during nanoparticle formulation, albumin may undergo conformational changes that alter its native structure, potentially compromising its interaction with key albumin receptors such as FcRn and gp60. To address this challenge and improve tumor accumulation, surface modifications by using coatings or targeting ligands have been explored [54, 55]. In this work, dextran-coated albumin nanoparticles were investigated as a strategy to enhance tumor targeting. Sagnella and coworkers suggested that interactions between dextran-based nanocarriers and the highly glycosylated surfaces of tumor cells may play a role in enhancing nanoparticle penetration [56].

Treatment with free bevacizumab combined with paclitaxel showed comparable outcomes to the formulation containing nanoencapsulated bevacizumab (B-NP-DEX) plus intravenous PTX in terms of tumor doubling time. Both treatment regimens extended the tumor doubling time by approximately 5 days compared to the control group (Fig. 2B). Moreover, both treatments significantly reduced tumor weight and decreased metabolic tumor volume, as demonstrated by PET imaging following ^18^F-FDG administration (Figs. 2C and 4D).

However, subtle differences indicated a superior therapeutic effect for the B-NP-DEX + PTX group than for the conventional BEVA + PTX one. Thus, animals treated with B-NP-DEX + PTX exhibited a greater reduction in TLG (Fig. 4D) and necrotic tumor areas (Fig. 5) than that observed in the group of animals treated with the combination between paclitaxel and free bevacizumab. In addition, NP-DEX + PTX showed a significant reduction in the tumor proliferation biomarker (ki67) and VEGF levels (Fig. 6), suggesting that the enhanced intratumoral accumulation of bevacizumab when delivered via nanoparticles may potentiate the antitumor response (Fig. 6D). The increased tumor concentration of bevacizumab is likely attributable to improved tumor penetration by the nanoparticles, facilitated by the enhanced permeability and retention (EPR) effect [57, 58], as evidenced by significantly higher bevacizumab levels (an 8-fold increase in the tumor-to-plasma ratio) when the antibody was nanoencapsulated compared to the combination between free bevacizumab and PTX (Fig. 3).

These findings suggest that bevacizumab encapsulated in dextran-coated albumin nanoparticles may enhance the therapeutic efficacy of paclitaxel. This effect may result from increased tumor accumulation of bevacizumab, potentially mediated by albumin-facilitated transport and dextran-related interactions. The subsequent vascular normalization and reduction in interstitial fluid pressure (IFP) could improve paclitaxel penetration and intratumoral distribution. Further mechanistic studies are needed to confirm these effects.

## Conclusions

The combination therapy between bevacizumab loaded in dextran-coated albumin nanoparticles combined with paclitaxel in a xenograft mouse model of colorectal cancer has demonstrated nuances of superior outcomes compared to free bevacizumab plus intravenous paclitaxel, mainly due to a better tumor penetration of the nanoencapsulated monoclonal antibody. Another important aspect related to the increased apparent concentration of bevacizumab in the tumor would be its potentially reduced distribution to healthy tissues, which could translate into a lower frequency and/or severity of treatment-related side effects. In any case, these findings highlight the potential of nanoparticle-based delivery platforms to enhance the therapeutic efficacy of antiangiogenic monoclonal antibodies and support further investigation into their role in augmenting chemotherapeutic responses.

## Supplementary Information

Below is the link to the electronic supplementary material.

Supplementary Material 1

## Data Availability

The datasets generated during and/or analysed during the current study are available from the corresponding author on reasonable request.
