# Supplementary material for: Evaluation of nanoencapsulated bevacizumab combined with paclitaxel in a colorectal cancer xenograft model
Source: Drug Deliv Transl Res. 2025 Aug 14;16(7):2194–206. doi: 10.1007/s13346-025-01941-6 (PMC13294262; doi:10.1007/s13346-025-01941-6)
Supplement: Supplementary file 1 — Supplementary Material 1 [file 13346_2025_1941_MOESM1_ESM.docx]

**Supplementary material**

***In vivo* efficacy study**

The following diagram (Figure 1S) summarizes the efficacy study in the animal model.


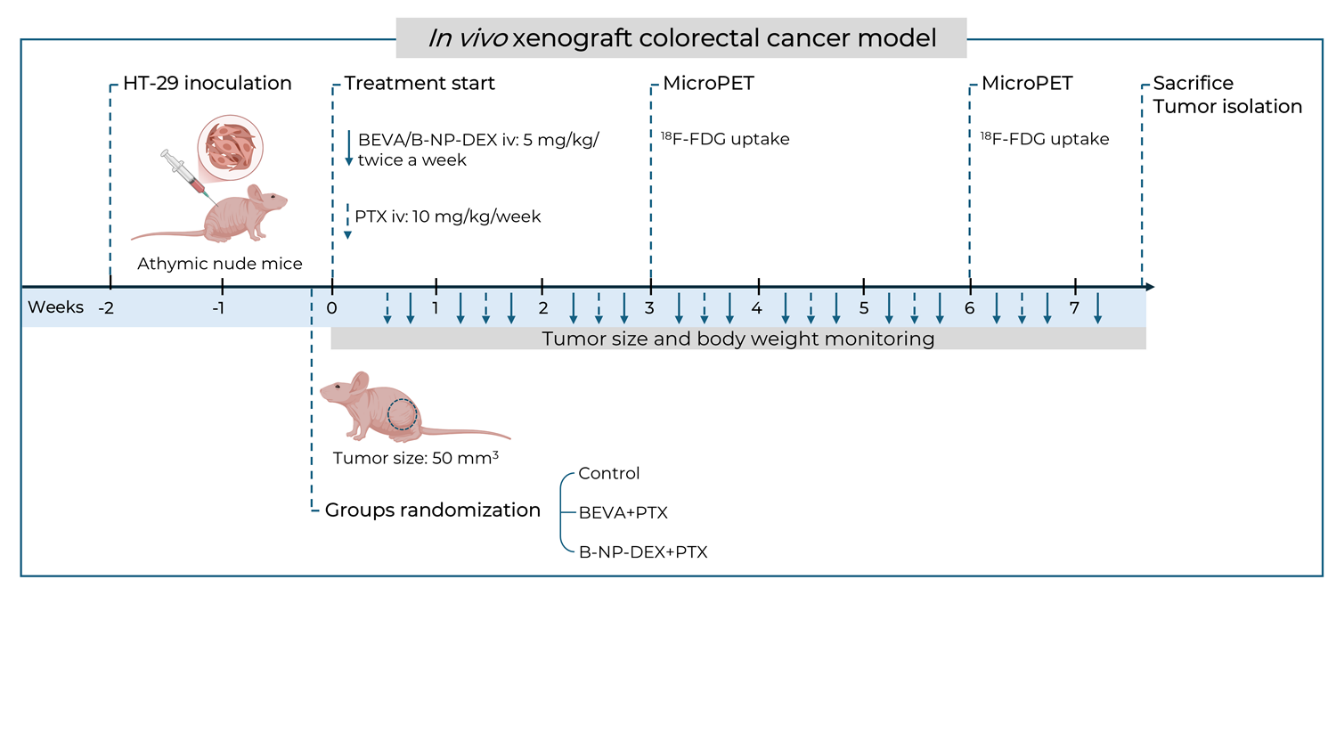


**Figure 1S.** Diagram summarizing the *in vivo* efficacy study.
